# BTK suppresses myeloma cellular senescence through activating AKT/P27/Rb signaling

**DOI:** 10.18632/oncotarget.18096

**Published:** 2017-05-23

**Authors:** Chunyan Gu, Hailin Peng, Yue Lu, Hongbao Yang, Zhidan Tian, Gang Yin, Wen Zhang, Sicheng Lu, Yi Zhang, Ye Yang

**Affiliations:** ^1^ The Third Affiliated Hospital, Nanjing University of Chinese Medicine, Nanjing 210023, China; ^2^ School of Medicine and Life Science, Nanjing University of Chinese Medicine, Nanjing 210023, China; ^3^ Department of Pathology, School of Medicine, University of Iowa, Iowa City, Iowa 52242, USA; ^4^ Department of Laboratory Medicine, Taizhou People's Hospital, Taizhou 225300, China; ^5^ Department of Radiation Oncology, Jiangsu Cancer Hospital, Nanjing 210009, China; ^6^ Center for New Drug Safety Evaluation and Research, China Pharmaceutical University, Nanjing 211198, China; ^7^ Department of Pathology, Nanjing First Hospital, Nanjing 210006, China; ^8^ Affiliated Hospital of Stomatology, Nanjing Medical University, Nanjing 210029, China; ^9^ Department of Burns and Plastic Surgery, Affiliated Hospital of Nantong University, Nantong 226001, China; ^10^ Key Laboratory of Acupuncture and Medicine Research of Ministry of Education, Nanjing University of Chinese Medicine, Nanjing 210023, China

**Keywords:** multiple myeloma, BTK, senescence, AKT, CGI-1746

## Abstract

We previously explored the role of BTK in maintaining multiple myeloma stem cells (MMSCs) self-renewal and drug-resistance. Here we investigated the elevation of BTK suppressing MM cellular senescence, a state of irreversible cellular growth arrest. We firstly discovered that an increased expression of BTK in MM samples compared to normal controls by immunohistochemistry (IHC), and significant chromosomal gain in primary samples. In addition, BTK high-expressing MM patients are associated with poor outcome in both Total Therapy 2 (TT2) and TT3 cohorts. Knockdown BTK expression by shRNA induced MM cellular senescence using β-galactosidase (SA-b-gal) staining, cell growth arrest by cell cycle staining and decreased clonogenicity while forcing BTK expression in MM cells abrogated these characteristics. We also validated this feature in mouse embryonic fibroblast cells (MEFs), which showed that elevated BTK expression was resistant to MEF senescence after serial cultivation *in vitro*. Further mechanism study revealed that BTK activated AKT signaling leading to down-regulation of P27 expression and hindered RB activity while AKT inhibitor, LY294002, overcame BTK-overexpression induced cellular senescence resistance. Eventually we demonstrated that BTK inhibitor, CGI-1746, induced MM cellular senescence, colony reduction and tumorigenecity inhibition *in vivo*. Summarily, we designate a novel mechanism of BTK in mediating MM growth, and BTK inhibitor is of great potential *in vivo* and *in vitro* suggesting BTK is a promising therapeutic target for MM.

## INTRODUCTION

Multiple myeloma (MM), the second most common blood cancer, is characterized by heterogenetic plasma cells clonal proliferation in the bone marrow microenvironment, monoclonal protein secretion in the blood or urine, anemia, bone lesion, hypercalcemia and renal damage [1, 2]. Despite the advanced development of novel chemo-therapies, like proteasome inhibitor and immunomodulator, Bortezomib and Lenalidomide even the newest generation Carfizomib and Pomalidomide combining with autologous stem cell transplant (ASCT) for MM treatment in recent years, patient responsiveness still varies greatly with most patients responding well to initial treatment, however a majority of patients become refractory to treatment and ultimately relapse [3–6], which makes MM remain an incurable disease. Increasing evidences showed sole tranditionally cytotoxic treatments, which focus on execution of cell death through cell apoptosis signal couldn't complecately eradicate the MM cells due to the exsitence of cancer stem cells and the cancer cell clonal evolution et al. [7–9]. Therefore, a further exploration of the genetically pathological mechanisms and inquiry for innovative therapeutic program on MM are matters that admit of no delay. In parallel with cellular apoptosis, cellular senescence which induces irreversible cell arrest through forcing the cells to quit the cell cycle suggests an alternative way to blunt MM growth and disable MM proliferation.

Hayflick and Moorhead firstly described cellular senescence (1961), and they found that after serial cultivation *in vitro* normal human fibroblasts entered a state of irreversible growth arrest [10], which now is discovered to be induced by DNA damage, cytotoxic drugs, intense oncogenic signaling, and telomere loss [11–14]. The senescent cells were identified in most types of cancer cells including MM. Consistent with its impact on cancer suppression, cellular senescence is mediated by several critical tumor-suppressor genes, the most crucial of which are P53 and RB [15–17]. Attractively, escalated studies demonstrated that senescence is prevalent in pre-malignant tumors, but progression to malignancy requires evading senescence [18] implying that senescence is an important tumor-suppressing mechanism that must be overcome during tumorigenesis. Thus, induction of cancer cellular senescence is considered to contribute to effectiveness of anticancer therapy by perturbing tumor growth [19].

One of X chromosome-localized Tec tyrosine kinases family genes named Bruton's tyrosine kinase (BTK), which is highly expressed in CD19^+^ B cells, CD14^+^ monocytes and B lymphoblasts, plays a central role in B-cell development and plasma cell differentiation [20, 21]. Functional disrupting mutations of BTK lead to X-linked agammaglobulinemia (XLA) in humans and X-linked immunodeficiency (Xid) in mice, primary immunodeficiency diseases which are characterized by lack of mature B cells and plasma cells and low levels of immunoglobulins [22, 23]. Interestingly, BALB/c.CBA/N mice carrying the defective BTK gene are resistant to pristine-induced plasmacytomagenesis indicating activation of BTK is essential for plasma tumor formation [24]. Intriguingly, elevated levels of BTK was reported as a poor prognosis marker in MM patients [25, 26]. Based on its role in development of B cells and its link to disease, BTK is an ideal therapeutic target of B cell malignancy. BTK inhibition has showed its potency in clinics as well as in clinical trials for Small Lymphocytic Lymphoma [27], Chronic Lymphocytic Leukemia [27], Diffuse large B-cell Lymphoma [28], and Mantle Cell Lymphoma [29]. Our previous study also revealed that BTK inhibitor CGI-1746 inhibits both clonogenic myeloma stem-like cells and bulk MM cells from primary patient samples and cell lines [30].

In this study, we disclosed the role of BTK in controlling MM cellular senescence using β-galactosidase (SA-b-gal) staining assay, cell cycle analysis and clonogenic examination, and verified this function in mouse embryonic fibroblast (MEF) cells. Furthermore, we demonstrated the mechanism under BTK-mediated MM senescence and showed CGI-1746, BTK inhibitor, induced MM cellular senescence *in vitro* and inhibited MM xenografted tumor formation *in vivo*, which not only highlight a novel feature of BTK in MM but also designate a promising therapeutic target with specific inhibitors.

## RESULTS

### Increased BTK expression correlates with poor survival in MM

**Figure 1:**
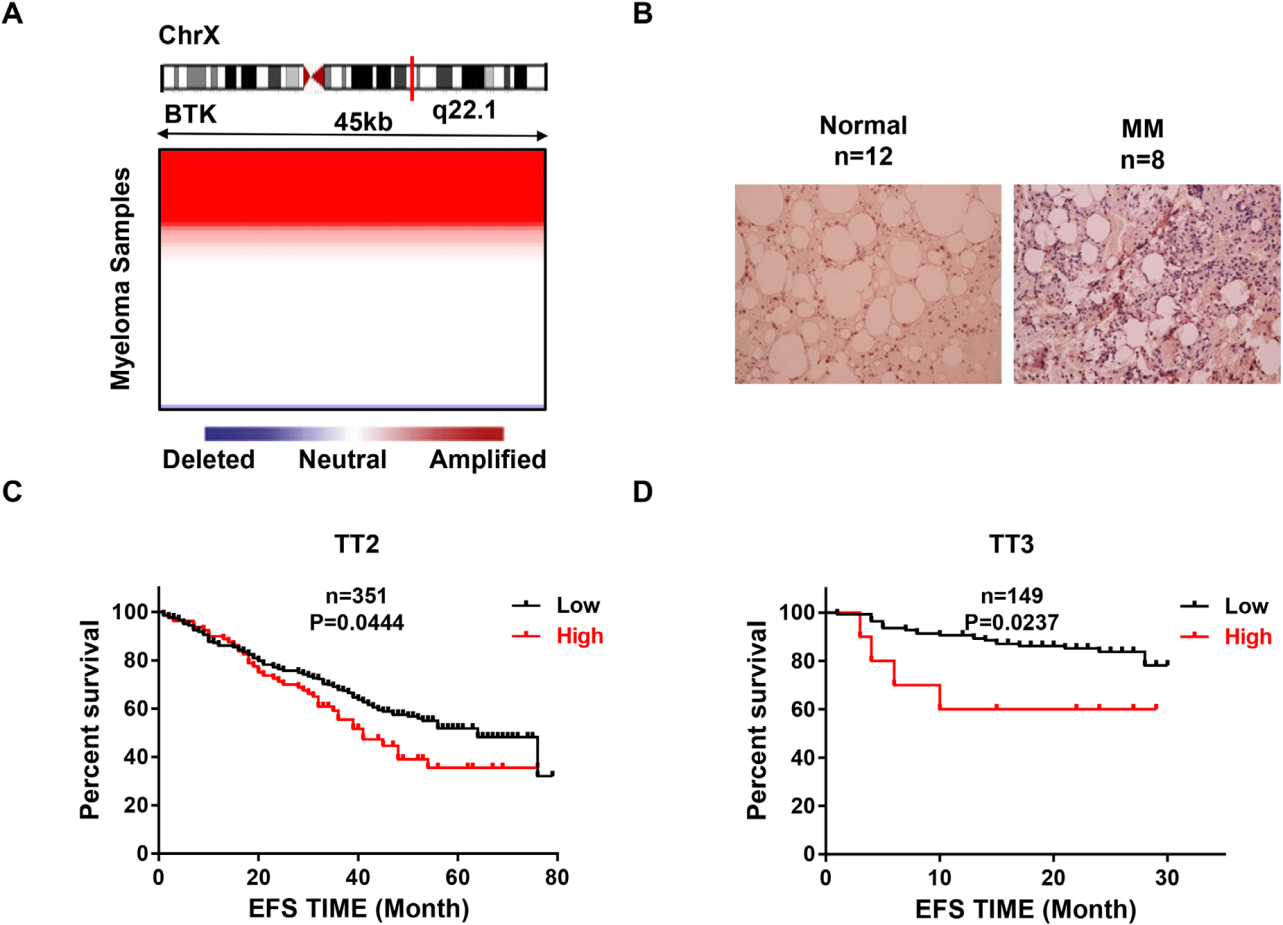
BTK is poor prognostic markers in MM **(A)** Heatmap illustrating BTK copy number variation in 67 primary MM samples. **(B)** Representative Immunohistochemistry staining on primary MM samples and normal controls (20X). **(C & D)** Kaplan-Meier analyses on the MM patients with high BTK expression had a significantly inferior event free survival in both TT2 **(C)** and TT3 **(D)** cohorts.

To evaluate the role of BTK in MM, We examined the array-based comparative genomic hybridization (aCGH) data obtained from 67 MM patients and the analysis revealed that the BTK locus is frequently amplified in MM patients (Figure 1A) [31]. We tested BTK expression in normal bone marrow samples (NP) and myeloma samples using immunohistochemistry (IHC) staining [32]. Impressively, BTK expression exhibited significant increase in MM samples compared to NP control (Figure 1B). In addition, the distinction between high and low BTK was of prognostic significance, as event-free survival (EFS) was reduced in MM patients bearing high BTK expression Total Therapy 2 (TT2) cohort (Figure 1C). A very similar fraction of BTK high-expressing myeloma and BTK–dependent reduction in survival was also observed in the Total Therapy 3 (TT3) cohort (Figure 1D). However, there is no significant difference (p>0.05) of BTK expression correlated with overall survival in TT serial cohorts (data not shown). Here we may propose that BTK is a poor prognostic marker in MM.

### Depletion of BTK in MM cells induces myeloma cellular senescence

**Figure 2:**
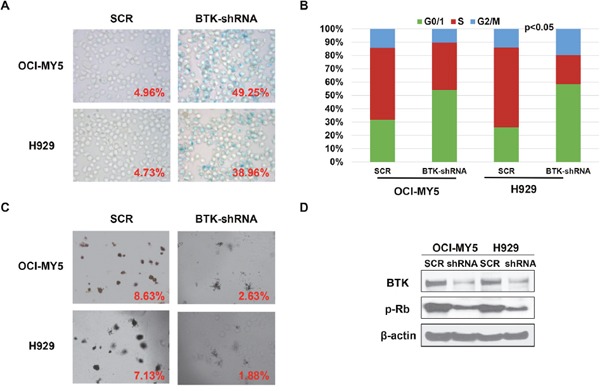
Depletion of BTK in MM cells induces myeloma cellular senescence **(A)** SA-b-galactosidase (Green) cellular senescence staining of OCI-MY5 and H929 MM cells transfected by BTK-shRNA *vs* scrambled sequence (SCR)-transfected control cells. **(B)** PI-staining cell cycle analysis of BTK-shRNA OCI-MY5 and H929 MM cells compared to SCR control cells. **(C)** Colony formation assay on BTK-shRNA OCI-MY5 and H929 MM cells compared to control cells. **(D)** Western blot on the BTK and pRB expression in the BTK-shRNA OCI-MY5 and H929 MM cells with SCR control cells.

Our previous study demonstrated that BTK is vital in maintaining MM cells self-renewal [30], while cellular senescence reflects a state of stable cell growth arrest suggesting that BTK may play an important role in MM cell senescence. SA-β-gal staining was employed to detect the cellular senescence states in H929 and OCI-MY5 BTK-shRNA cells. MM cells transfected with non-targeting scramble sequences serve as control. As shown in Figure 2A, substantial SA-β-gal positive cells were found in BTK-silenced MM cells, while bare positive cells appeared in control cells. Since cellular senescence is characterized by cell growth arrest, we further examined the effect of BTK on cell cycle. As seen in Figure 2C, decreased BTK expression in MM cells led to G0/G1 arrest showing as increased G0/G1 cell population and corresponding reduction of S and G2/M fraction. Clonogenic formation assay validated these results and showed that MM cells with BTK depletion generated a much lower colony numbers than the control cells (Figure 2B). To query how BTK prompts MM senescence resistance, western blot was performed and found that the key senescence regulator phosphorated-RB (pRB) was reduced in BTK-shRNA cells relative to control cells (Figure 2D). The results presented in this figure provided the preliminary evidence for a role of BTK suppressing myeloma cell senescence.

### Elevation of BTK expression is resistant to cellular senescence

**Figure 3:**
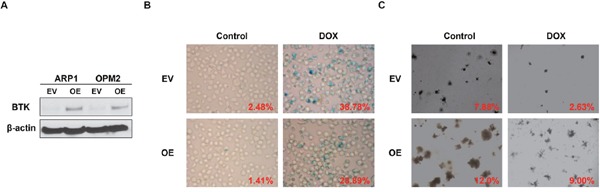
Increased BTK expression suppresses doxorubicin-induced cellular senescence in MM cells **(A)** Western blot analysis for the ARP1, OPM2 MM cells transfected by BTK-cDNA (OE) *vs* empty vector (EV)-transfected control cells. **(B & C)** SA-b-galactosidase (Green) cellular senescence staining **(B)** and colony formation assay **(C)** of OPM2 BTK-OE and EV in the presence or absence of Doxorubicin (30 nM).

To further prove that BTK is the driver for MM senescence resistance, we overexpressed BTK expression (OE) in APR1 and OPM2 cells by lentivirus, and western blot examination verified the heightened of BTK expression in the BTK-OE cells compared to the empty vector (EV) transfected control cells (Figure 3A). The OPM2 EV and OE cells were treated with doxorubicin (30 nM) for 48 hours, then stained for SA-β-gal activity. Consistent with our speculation, overexpressed BTK repressed doxorubicin-induced cellular senescence in MM cells compared to EV controls (Figure 3B). Following colony formation assay confirmed BTK function in senescence and exhibited that OPM2-OE generated much more colonies than EV cells when plating the same number of cells initially (Figure 3C).

**Figure 4:**
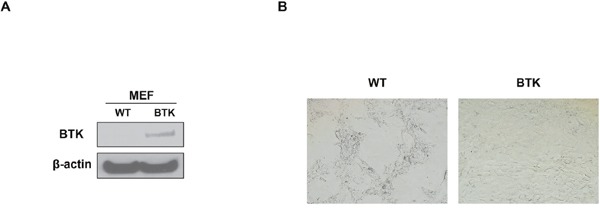
BTK suppresses cellular senescence in mouse embryonic fibroblasts **(A)** Western blot on the expression level of BTK expression in BTK-OE and WT MEFs. **(B)** SA-b-galactosidase (Green) staining was performed on MEFs transfected with BTK-cDNA or empty vector and passaged for 5 generation.

To affirm the role of BTK in cellular senescence, MEF cells, the best tool to evaluate cell senescence, was introduced into our study. We overexpressed murine BTK cDNA in MEF cells derived from C57BL/6 mice, and western blot assay proved the increase of BTK in cDNA transfection (OE) group compared to the none transfection (WT) control cells (Figure 4A). SA-β-gal staining indicated that there were less senescent cells in BTK-OE MEFs than control MEFs at the same generation (Figure 4B). Thus it is plausible for us to conclude that BTK suppresses MM cellular senescence.

### BTK activates ATK signaling in MM cells

**Figure 5:**
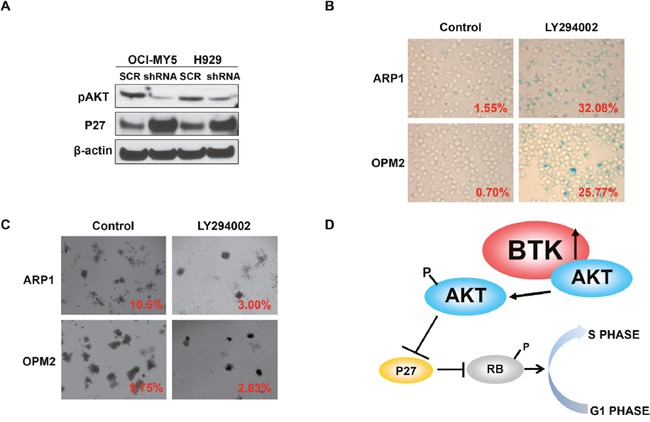
BTK activates AKT and blocks RB activity in MM cells **(A)** Western blot analysis on pAKT and P27 expression in BTK-shRNA and control OCI-MY5 and H929 cells. **(B & C)** SA-b-galactosidase cellular senescence staining **(B)** and colony formation assay **(C)** of OPM2 BTK-OE treated with or without AKT inhibition, LY294002. **(D)** The working model of BTK inducing MM cellular senescence.

Our previous study demonstrated that BTK directly bound and phosphorylated AKT in MM cells leading to AKT signaling activation [30]. To further explore the mechanism of BTK-mediated anti-senescence in MM, we proved the interaction between BTK and AKT using immunofluorescence stain in which BTK labeled with green color and AKT conjugated with red color co-localized with each other in both OCI-MY5 and H929 MM cells (data not shown). Western blot assay showed that down-regulation of BTK decreased pAKT (S473), and increased P27, target of AKT, in both OCI-MY5 and H929 MM cells (Figure 5A). To validate that AKT is the major signaling for BTK-mediated senescence, AKT inhibitor, LY294002, was introduced to testify if prevention of AKT activation could block anti-senescent function of BTK. As expected, LY249002 overcame BTK overexpression induced anti-senescence effect and exerted profound senescent cells after treatment for 48 in ARP1 BTK-OE cells (Figure 5B). Colony formation experiment illustrated a consistent result that AKT inhibition blocked the long-tern cellular self-renewal of both ARP1 BTK-OE cells (Figure 5C), which showed potential resistance to doxorubicin in above study. Overexpression of BTK in OPM2 cells showed similar anti-senescent effect in both SA-β-gal staining and clonogenic assay (data not shown). These findings suggest that BTK mediates MM cellular senescence through activating AKT/P27/RB signaling (Figure 5D).

### BTK inhibitor, CGI-1746, induces MM cellular senescence and impedes MM xenograft tumor growth *in vivo*

**Figure 6:**
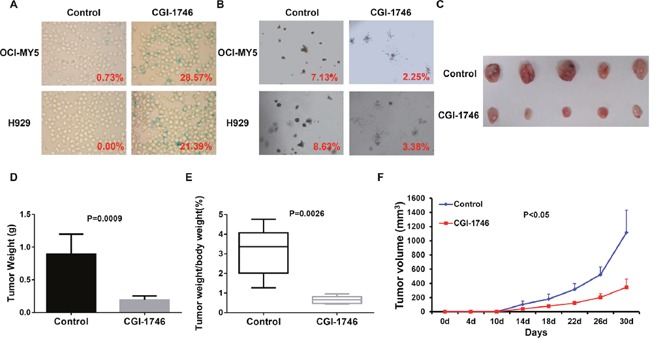
BTK inhibitor, CGI-1746, showed potent therapeutic effect on MM *in vitro* and *in vivo* **(A & B)** SA-b-galactosidase staining **(A)** and colony formation assay **(B)** on OCI-MY5 and H929 MM cells treated in absence or presence of CGI-1746. **(C)** Tumors dissected from NSG mice treated with or without CGI-1746. **(D & E)** Mean weight of tumor **(D)** and ratio of tumor to body weight **(E)** from OCI-MY5 xenograft mice received with CGI-1746 treatment compared to non-treatment control mice. **(F)** Time course of tumor growth in NOD/SCID mice received OCI-MY5 cell with or without CGI-1746 treatment (n=5).

To extend our findings into pre-clinics study, we examined the effect of BTK inhibitor, CGI-1746, on MM senescence. Intriguingly, CGI-1746 treatment resulted in considerable MM cellular senescence in OCI-MY5 and H929 cells (Figure 6A) and restrained MM clonogenicity (Figure 6B), which reflected the lengthy cell growth. To testify CGI-1746 *in vivo*, we xenografted OCI-MY5 cells subcutaneously into NSG mice respectively (n = 5), CGI-1476 treatment was start 7 days after injection. Tumor diameter was measured twice per week to evaluate the tumor growth rate. After 30 days, the tumors from control group, PBS Treatment, were visibly smaller than their counterparts (Figure 6C). The average weight of control tumors and the ratio of tumor weight to body weight were higher than the treatment tumors (Figure 6D & 6E). Time course analysis of tumor growth demonstrated that CGI-1746 outstandingly lagged the MM tumor growth *in vivo* (Figure 6F).

## DISCUSSION

Multiple myeloma is an incurable plasma cells malignancy, which typically responds to current treatment, eventually relapses and leads to patients’ death finally. Therefore, identification of novel therapeutic markers and development of innovative treatment strategy brook no delay. Interestingly, IHC staining and CGH array revealed that BTK increased in MM cells compared to normal control cells, moreover, high BTK-expressing patients are associated with poor outcome in TT2 and TT3 cohorts suggesting that BKT may be a potential therapeutic target for MM. Displaying exciting clinical outcome of BKT inhibitor, ibrutinib, in patients with mantle cell lymphoma (MCL) and chronic lymphocytic leukemia (CLL) [33–35], ibrutinib and other BTK inhibitors are now undergoing clinical testing for multiple myeloma, yet important questions on the role of BTK in myeloma biology and treatment are still outstanding. In this study, we demonstrated that down-regulation of BTK by shRNA induced MM cellular senescence, which was featured by positive SA-β-gal staining, cell growth arrest by cell cycle analysis and reduction of colony formation capability of MM cells. This is a novel account for BTK mediating MM cell growth. We further overexpressed BTK by transfecting BTK cDNA into MM cells and MEF cells respectively. Compatible with MM cellular senescence caused by BTK deduction, forcing BTK expression was resistant to senescence induced by doxorubicin treatment or serial cell passages. Now we can add the role of senescent suppressor into the functional lists of BTK.

X-linked agammaglobulinemia (called X-linked hypogammaglobulinemia, XLA, Bruton type agammaglobulinemia as well) is characterized by mutation occurring at the Bruton's tyrosine kinase gene that leads to a severe block in B cell development from the pro-B to pre-B cell stage and a reduced immunoglobulin production in the serum. However, how BTK blocks B cells maturation is still unknown and our study provide a possible explanation for that B cells development may be hindered by loss function of BTK induced cellular senescence. CBA/CaHN-*Btk*^xid^/J mice with have a mutated version of the mouse BTK gene, are the mouse model of XLA in human, which exhibit a similar, yet around 50% milder B cells deficiency while XLA account for nearly 90% [23, 36]. Masahiro Shinohara et al. reported that BTK synergized TEC regulating osteoclast differentiation in mice [37], which suggested that other TEC genes including TEC may compensate for the loss function of BTK leading to a milder B cells deficiency in mice while BTK may be the non-substitutable factor for B cell development in human. This makes BTK acting as an ideally potential therapeutic target for B cell malignancy.

Our mechanistic study demonstrated that BTK directly interacted with AKT and increased pAKT, an activated form of AKT. AKT signaling is an important regulator of cell homeostasis and its disregulation is associated with solid tumor and hematological cancers. Activation of AKT specifically phosphorylates P27 at Ser10 site, which promotes P27 binding to 14-3-3 and cytoplasmic localization, a non-functional form of P27 [38, 39]. In addition, Ser10 phosphorylation of P27 is also a prerequisite for its degradation via the E3 ubiquitin ligases SKP2 (nuclear) and KPC (cytoplasmic), respectively [40]. P27 is a broad spectrum of cyclin-dependent kinases (CDKs) inhibitor. In a variety of malignancies, suppression of P27 prompts Cdk4/6-cyclinDs complex-mediated RB phosphorylation and inactivation of RB (pRB) allows the transcription of E2F-dependent various cell cycle regulatory genes, inducing cell proliferation and suppressing cellular senescence [17, 41]. In this study, we found that knockdown of BTK decreased pAKT, and increased P27 expression in MM cells by western blot. Therefore, we summarized that BKT suppresses MM senescence through activating AKT/P27/pRB signaling (Figure 6E).

Since there are major two regulator for cellular senescence, RB and P53, we further explored if P53 is involved in BTK induced MM senescence. Our study illustrated that increase of BTK expression activated AKT in ARP1 and OPM2 MM cells and was resistant to doxorubicin treatment induced cell senescence. Since OPM2 MM cells harbor P53 mutation and ARP1 cells are no P53 expression, we proposed that BTK mediated MM cellular senescence independent of P53.

Finally, we showed that CGI-1746, a novel and potent BTK inhibitor, could induce MM cellular senescence and inhibit MM cells colony formation *in vitro* and reduce xenografted tumor derived from MM cell lines *in vivo*.

Collectively, our findings exhibit a novel and mechanistic insight into the function of BTK on suppressing MM senescence both *in vitro* and *in vivo*, and highlight BTK as potential therapeutic target to cure MM.

## MATERIALS AND METHODS

### Cell lines and cell culture

Human MM cell lines, APR1, OPM2, OCI-MY5 and H929, were cultured in RPMI 1640 medium (Gibco, Grand Island, NY) supplemented with 1% penicillin and streptomycin (P/S) solution (100 μg/mL, Sigma, St. Louis, MO) and 10% fetal bovine serum (FBS) (Gibco), in 5% CO2 at 37°C. Mouse embryonic fibroblasts (MEFs) were purchased from Amsbio LLC (Cambridge, MA). MEFs and HEK-293T were cultured in DMEM medium containing 10% FBS and 1% P/S solution in humidified 95% air and 5% CO2 at 37°C.

### Reagents

Senescence β-Galactosidase Staining Kit (Catalog number:# 9860), AKT (Catalog number:#9272 & #9271), RB (Catalog number:#9969), and β-ACTIN (Catalog number:#4967) were obtained from Cell Signaling Technology (Danvers, MA). P27 (Catalog number: sc-528), BTK (Catalog number: sc-1108) antibody purchased from Santa Cruz Biotechnology (Dallas, Texas). CGI-1746 was provided by Good East Pharmaceutical Technology Yangzhou Co.Ltd. Doxorubicin and doxycycline hyclate were purchased from Sigma. Propidium Iodide and RNase A stock solution were from Invitrogen (Grand Island, NY).

### Senescence β-galactosidase staining

MM cells β-Galactosidase staining was performed according to the protocol. Briefly, around 1,000,000 MM cells were fixed for 20 mins, then rinsed with 1X PBS solution for two times, and the cells were incubated in 2 ml of the β -Galactosidase Staining Solution overnight in a dry incubator at 37°C.

### Cell cycle analysis

Cell cycle was analyzed by Propidium Iodide (PI) staining. Briefly, 1,000,000 cells were fixed with 2 ml cold ethanol for 1 hour at 4°C. After washed with ice-cold PBS for two times, the cells were suspended with 1 ml of PI staining solution (40 μg/ml in PBS), supplemented with 50 μl of RNase A stock solution (10 μg/ml) and incubated 2 hour at 4°C. Then cell cycle was analyzed using FACSScan flow cytometer (Becton Dickinson, San Jose, CA).

### Soft agar clonogenic assay

Clonogenic formation was performed by plating 10,000 MM cells in 0.5 mL 0.33% agar in 12-well plate. The cells were incubated at 37°C with 5% CO_2_ and fed by RPMI 1640 medium with 10% FBS twice for the first week, and then treated medium containing doxycycline or doxorubicin for another 1 week. The colonies were imaged and colony numbers were calculated using Image J.

### Western blots

Western blots were used to measure the protein levels in MM cells. Briefly, cells were lysed in Mammalian Cell Extraction Kit (Catalog number: K269-500, Biovision, Milpitas, CA). Around 10 μg protein per well was loaded to SDS-PAGE using 4%-12% polyacrylamide gels prior to the PVDF membrane transfer. PVDF membrane was blocked with 5% non-fat dry milk in Tris buffered saline (TBS) containing 0.05% Tween-20 (TBST), and then incubated with primary antibodies overnight at 4°C. Western were visualized with secondary antibodies conjugated with HRP and SuperSignal West Pico (Pierce, Rockford, IL). Membranes were stripped and re-probed for β-ACTIN as controls.

### Gene expression profiling (GEP) and data analysis

GEP, using the Affymetrix U133 Plus2.0 microarray, was performed as previously described [32, 42, 43].

### Lentivirus expression vector system

Lentiviral based gene silencing and overexpression constructs were performed as previously described [32, 44]. BTK-shRNA double-stranded oligonucleotides were cloned into TRIPZ vector and BTK cDNA was cloned into pCDH vector system. Recombinant lentivirus was produced using transient 293T cell transfection. Transduction efficiency was determined by flow cytometry with 95% efficiency.

### A xenograft myeloma mouse model

All animal work was performed according to the guidelines of the Institutional Animal Care and local veterinary office and ethics committee of the University of IOWA, USA (IACUC 1301010) under approved protocol. MM cells (1 × 10^6^) were subcutaneously xenografted into both flank of 6-8 weeks’ NOD. Cg-Rag1 (NSG) mice (Jackson laboratory, Bar Harbor, Maine) (n = 5). After 7 days, CGI-1746 (200 mg/kg, SC) treatment was started and injected daily. Tumor burdens were measured by tumor volume. Once tumors reached 20 mm in diameter, mice were sacrificed by CO_2_ asphyxiation.

### Statistical analysis

The MM patients’ survival data were plotted by Kaplan-meier curve and analyzed using log-rank test. All other values were analyzed by two-tailed Student's t-test and expressed as mean ± SD. A p<0.05 was considered as significant.
